# Association between the nucleosome footprint of plasma DNA and neoadjuvant chemotherapy response for breast cancer

**DOI:** 10.1038/s41523-021-00237-5

**Published:** 2021-03-26

**Authors:** Xu Yang, Geng-Xi Cai, Bo-Wei Han, Zhi-Wei Guo, Ying-Song Wu, Xiaoming Lyu, Li-Min Huang, Yuan-Bin Zhang, Xin Li, Guo-Lin Ye, Xue-Xi Yang

**Affiliations:** 1 Clinical Innovation and Research Center, Shenzhen Hospital, Southern Medical University, Shenzhen, China; 2 The Third School of Clinical Medicine, Southern Medical University, Guangzhou, China; 3 Department of Breast Surgery, The First People’s Hospital of Foshan, Foshan, China; 4 Sun Yat-Sen Memorial Hospital, Sun Yat-Sen University, Guangzhou, China; 5 Institute of Antibody Engineering, School of Laboratory Medicine and Biotechnology, Southern Medical University, Guangzhou, China; 6 Department of Laboratory Medicine, The Third Affiliated Hospital, Southern Medical University, Guangzhou, China

**Keywords:** Tumour biomarkers, Breast cancer

## Abstract

Gene expression signatures have been used to predict the outcome of chemotherapy for breast cancer. The nucleosome footprint of cell-free DNA (cfDNA) carries gene expression information of the original tissues and thus may be used to predict the response to chemotherapy. Here we carried out the nucleosome positioning on cfDNA from 85 breast cancer patients and 85 healthy individuals and two cancer cell lines T-47D and MDA-MB-231 using low-coverage whole-genome sequencing (LCWGS) method. The patients showed distinct nucleosome footprints at Transcription Start Sites (TSSs) compared with normal donors. In order to identify the footprints of cfDNA corresponding with the responses to neoadjuvant chemotherapy in patients, we mapped on nucleosome positions on cfDNA of patients with different responses: responders (pretreatment, *n* = 28; post-1 cycle, post-3/4 cycles, and post-8 cycles of treatment, *n* = 12) and nonresponders (pretreatment, *n* = 10; post-1 cycle, post-3/4 cycles, and post-8 cycles of treatment, *n* = 10). The coverage depth near TSSs in plasma cfDNA differed significantly between responders and nonresponders at pretreatment, and also after neoadjuvant chemotherapy treatment cycles. We identified 232 TSSs with differential footprints at pretreatment and 321 after treatment and found enrichment in Gene Ontology terms such as cell growth inhibition, tumor suppressor, necrotic cell death, acute inflammatory response, T cell receptor signaling pathway, and positive regulation of vascular endothelial growth factor production. These results suggest that cfDNA nucleosome footprints may be used to predict the efficacy of neoadjuvant chemotherapy for breast cancer patients and thus may provide help in decision making for individual patients.

## Introduction

Noninvasive tests offer a number of compelling advantages, and liquid biopsies have been developed as a valuable tool over the past decade, in particular for chromosomal aneuploidy screening and companion diagnostic testing. Blood is generally the easiest specimen type to work with. In peripheral blood, testing may target circulating tumor cells; circulating cell-free DNA (cfDNA), which in cancer patients contains circulating tumor DNA (ctDNA); circulating cell-free RNA (cfRNA); or circulating extracellular vesicles (EVs), such as exosomes, tumor-educated platelets, proteins, and metabolites^1,2^. The concentration of cfDNA is relatively high and stable in blood, and cfDNA has therefore become a widely used analyte in liquid biopsy. cfDNA is derived mainly from apoptotic and necrotic cells of primary tumors, circulating tumor cells, and normal cells^3,4^ and is usually bound to mononucleosomes rather than present as free DNA^1,5^. ctDNA makes up only a small proportion of the total plasma cfDNA^6^, requiring a large volume of plasma and sensitive detection methods, and cannot be used to detect cancer when there is a low ctDNA:cfDNA concentration ratio or no mutation^7^.

In eukaryotes, nucleosomes are repeating units of chromatin that are thought to strongly affect gene expression^8,9^. A nucleosome-free region (NFR) or a nucleosome-depleted region (NDR) is usually present in the transcriptionally active core region of the gene promoter^9^. Nucleosome positioning relative to transcription start sites (TSSs) is directly correlated with RNA polymerase II (Pol II) binding, and genome-wide maps exhibit differential nucleosome positioning in active and silent genes^10^. Nucleosomes consist of 145–147 bp DNA segments wrapped around a histone octamer composed of two molecules each of the four core histone proteins (H2A, H2B, H3, and H4), cemented to the nucleosome surface by an additional ~20 bp DNA (linker DNA)^11^. cfDNA fragments are around 166 bp in length^12^, which corresponds to the nucleosome DNA plus linker DNA.

In previous studies^13,14^, deep genome-wide sequencing of circulating cell-free DNA enabled identification of maps of nucleosome occupancy that provide a direct footprint of transcription factor occupancy. In addition, nucleosome footprint patterns in cell-free DNA are often specific to a type of cancer^13^. The presence or absence of nucleosomes in the TSS region of cfDNA results from expressed or silent genes in origin tissue and thus can be used to predict gene expression. Peter et al. determined that several TSSs matched with the expressed isoforms of genes from metastatic primary tumors^14^, and this result has been confirmed in individuals of different ages^15^.

Conventional gene expression profiling may be used to predict prognosis and guide treatment in the early stages of breast cancer. Five multigene expression testing techniques were included in the guidelines for breast cancer published by the National Comprehensive Cancer Network^16^ and the American Joint Committee on Cancer^17^ in 2018: the Oncotype DX 21-gene assay, the Mamma Print 70-gene assay, the Endo-Predict 12-gene assay, and the PAM 50 (Prosigna) and Breast Cancer Index tests. However, these tests are usually performed using tissue biopsies, which require invasive surgery and cannot capture the entire genomic landscape of breast tumors.

In the present study, to explore relationships among the cfDNA nucleosome profile, intracellular nucleosome positioning, and gene expression, we used breast cancer cell line supernatant to mimic plasma cfDNA and sequenced it using next-generation sequencing technology. Simultaneously the cell particles were subjected to MNase sequencing and mRNA sequencing. We analyzed correlations by comparing the nucleosome footprint profiles 1 kb upstream and 1 kb downstream of TSSs, in particular at the exact positions of TSSs. Furthermore, plasma cfDNA footprint profiles were characterized by low-coverage sequencing, and differences in profiles in TSS-adjacent regions were analyzed between healthy individuals and patients and between responders and nonresponders to neoadjuvant epirubicin-cyclophosphamide-docetaxel chemotherapy. Finally, we used plasma collected before and after treatment to assess correlations between cfDNA footprint profiles and response to breast cancer treatment and to examine changes in the footprint associated with treatment.

## Results

**Fig. 1:**
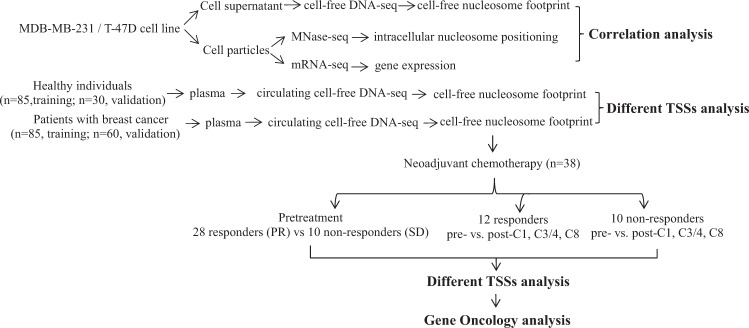
Experimental design and flowchart of data analyses. The correlation of cfDNA nucleosome profile, intracellular nucleosome positioning, and gene expression was analyzed by using breast cancer cell lines MDA-MB-231 and T-47D. Next the different nucleosome positioning on cfDNA of breast cancer patients and healthy individuals was analyzed. And finally the footprints of cfDNA corresponding with the responses to neoadjuvant chemotherapy in patients at pretreatment and after teatment were identified.

A study flowchart that includes analytical methods is shown in Fig. 1.

### The cfDNA nucleosome footprint reveals intracellular nucleosome positioning and gene expression

**Fig. 2:**
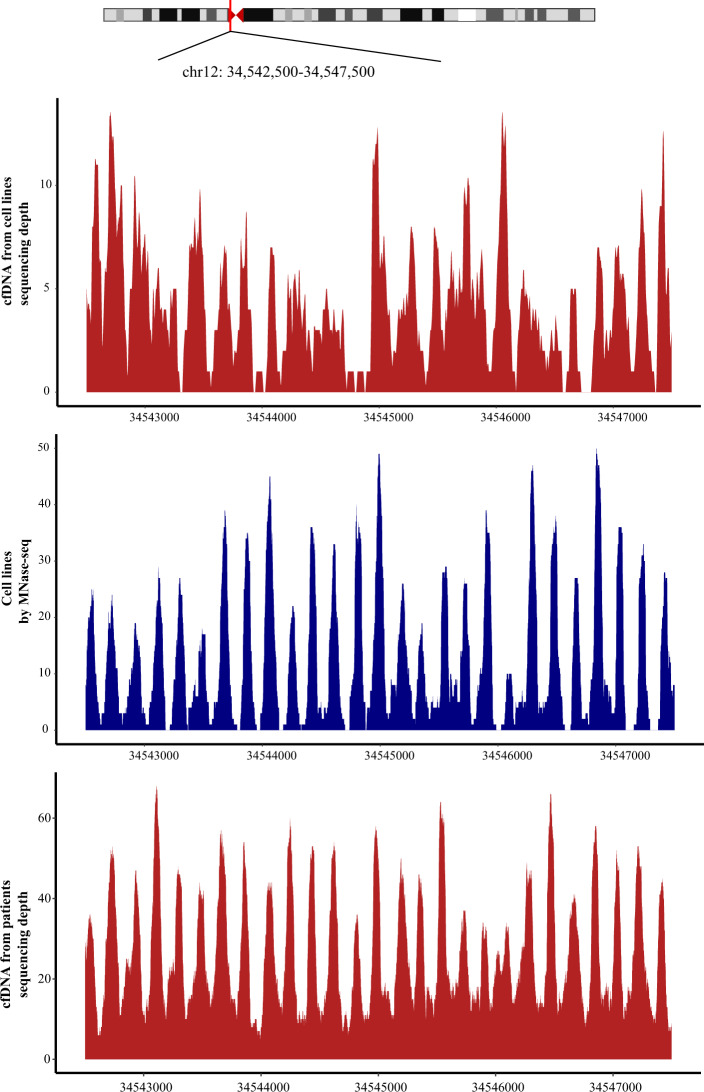
Genome browser view in chromosome 12 with enlargement of 12p11.1. CfDNA-seq data from the MDA-MB-231 and T-47D cell lines (up), MNase-seq data from the MDA-MB-231 and T-47D cell lines (mid), and circulating cell-free DNA from breast cancer patients (down). The region contains an extreme example of sequence-directed nucleosome positioning. Read depth coverage for cfDNA-seq data from cell lines (*n* = 2; merged data) and patients (*n* = 50; merged data) is shown in red, and the MNase midpoint density map from cell lines cell lines (*n* = 2; merged data) is shown in blue.

To determine whether the cfDNA profile reflected intracellular nucleosome positioning and predicted gene expression, we performed cfDNA whole-genome sequencing of cell supernatant as well as MNase sequencing and mRNA sequencing of the MDA-MB-231 and T-47D cell lines, respectively. We analyzed the cfDNA sequencing library using a 2100 Bioanalyzer, and the lengths of the inserted DNA fragments from cell supernatant and from the cell genome digested by MNase were ~166 bp and ~146 bp, respectively (ligation to ~90 bp adapter DNA; Supplementary Fig. S1), which is consistent with previous reports^14^. Next we analyzed chromosome 12p11.1, a 76 kb region containing more than 400 nucleosomes with strong positioning properties by cfDNA-seq and MNase-seq for cell line supernatant and plasma cfDNA from 50 breast cancer patients. The cfDNA read depth map showed a crest pattern whose position was highly correlated with that found in the MNase map, in particular in plasma DNA (Fig. 2).

**Fig. 3:**
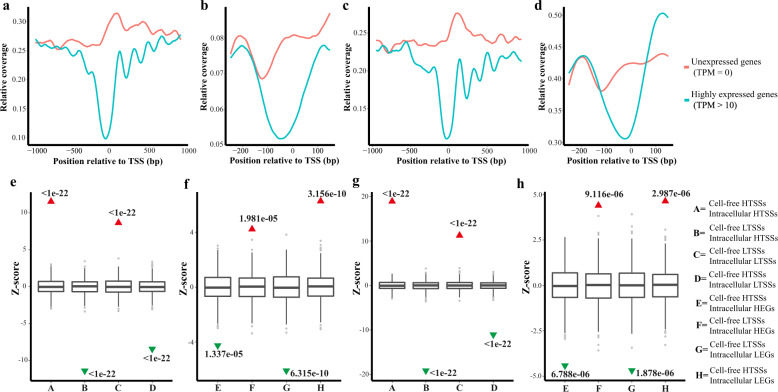
The cfDNA nucleosome footprint of cell supernatant reveals intracellular nucleosome positioning and gene expression. DNA read depth maps of the TSSs by MNase-seq for highly expressed genes (TPM > 10, blue) and unexpressed genes (TPM = 0, red) in the MDA-MB-231 cell line (**a**) and T-47D cell line (**b**) and cfDNA-seq for highly expressed genes (TPM > 10, blue) and unexpressed genes (TPM = 0, red) in the MDA-MB-231 cell line (**c**) and T-47D cell line (**d**). Overlaps of DTSSs (different high coverage depths around TSSs) in cfDNA-seq and MNase-seq of the MDA-MB-231 cell line (**e**) and T-47D cell line (**f**) and DTSSs by cfDNA-seq and DEGs (differentially expressed genes) matched by mRNA-seq of the MDA-MB-231 cell line (**g**) and T-47D cell line (**h**). Box plots represent the expected null distribution of overlaps from 1000 permutations (two sided, *p* values computed using a standard normal distribution). The extremes of the boxes define the upper and lower quartiles, and the center lines define the median. Whiskers indicate 1.5 times the interquartile range (IQR). Triangles represent observed overlap (red if significantly enriched, green if significantly depleted).

We also screened highly expressed genes (TPM > 10) and unexpressed genes (TPM = 0) in these two cell lines using mRNA-seq, then analyzed the genes’ sequence coverage depth around TSSs using MNase-seq and cfDNA-seq. The results of MNase-seq showed that the sequence coverage depth around TSSs was significantly lower for highly expressed genes than for unexpressed genes (Fig. 3a, b). Analyses of the matching sequence coverage depth around TSSs of the cfDNA showed the same phenomenon, with a significant decrease in coverage depth at the TSS site (Fig. 3c, d).

Permutation tests to estimate the overlaps between cell-free HTSSs (high coverage depth around TSSs) or LTSSs (low coverage depth around TSSs) and intracellular HTSSs or LTSSs revealed significant enrichment for HTSSs pairs (*p* < 10^−22^) and LTSSs pairs (*p* < 10^−22^). This significant enrichment was not observed in overlaps between cell-free HTSSs and intracellular LTSSs or cell-free LTSSs and intracellular HTSSs (Fig. 3e, g). These findings suggest that cfDNA-seq of the cell supernatant can reveal intracellular nucleosome positioning. Similar permutation tests were performed to estimate the enrichment of overlaps between cell-free HTSSs or LTSSs and HEGs (highly expressed genes) or LEGs (lowly expressed genes). The results revealed a pattern opposite to that found between cell-free and intracellular TSSs. The LTSSs of cfDNA were highly consistent with the corresponding HEGs (*p* = 1.981 × 10^−05^), and the HTSSs of cfDNA were highly consistent with the LEGs (*p* = 3.156 × 10^−10^). However, this phenomenon was not observed in the HTSSs of cfDNA and the corresponding HEGs or in the LTSSs of cfDNA and the corresponding LEGs (Fig. 3f, h).

### Breast cancer patients’ cfDNA coverage is related to gene expression in breast cancer cells

**Fig. 4:**
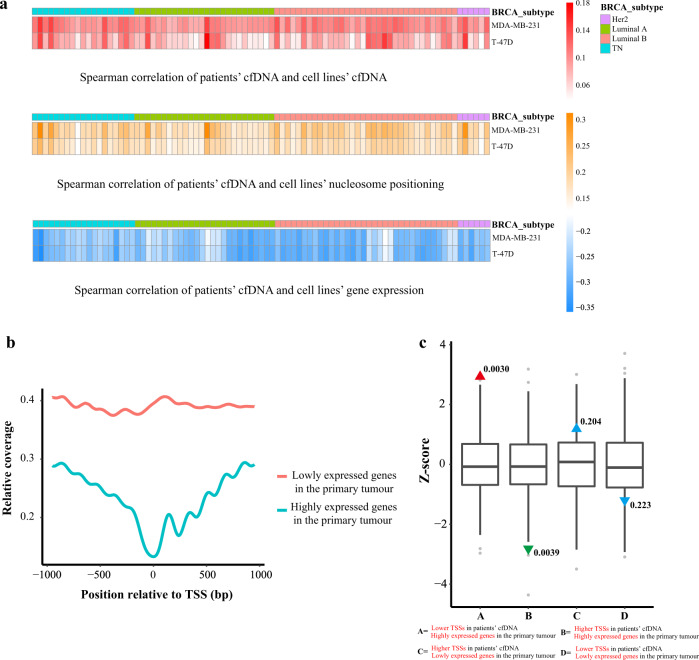
Breast cancer patients’ cfDNA coverage is related to gene expression in breast cancer cells. **a** Correlations between DTSSs by circulating cell-free DNA-seq from 85 breast cancer patients and DEGs matched by mRNA-seq, DTSSs by MNase-seq, and DTSSs by cell-free DNA-seq in the MDA-MB-231 cell line and T-47D cell line. **b** Circulating cell-free DNA-seq read depth maps of promoter regions from 85 breast cancer patients for highly expressed genes (TPM > 10, blue) and unexpressed genes (TPM = 0, red) in the MDA-MB-231 cell line and T-47D cell line. **c** Overlaps of coverage depth around TSSs of expressed genes in primary tumor tissue compared to adjacent breast tissue (TCGA) in breast cancer patients (*n* = 85) compared to healthy donors (*n* = 85). Box plots represent the expected null distribution of overlaps from 1000 permutations (two sided, *p* values com*p*uted using a standard normal distribution). The extremes of the boxes define the upper and lower quartiles, and the center lines define the median. Whiskers indicate 1.5 times the interquartile range (IQR). Triangles represent observed overlap (red if significantly enriched, green if significantly depleted).

We sequenced the circulating cell-free DNA from plasma collected from 85 healthy individuals and 85 breast cancer patients and compared it to cfDNA collected from the supernatant of the breast cancer cell lines. Correlation analyses showed that the gene sequence coverage depth near cfDNA TSSs in cell lines was positively correlated with nucleosome positioning assessed by MNase-seq and negatively correlated with gene expression assessed by mRNA-seq (Fig. 4a). The cfDNA pattern from 85 breast cancer patients was the same as that of the two breast cancer cell lines (Fig. 3a–d). It is interesting that this pattern was more obvious in the cfDNA from breast cancer patients, with a lower high-expression gene sequence coverage depth near TSSs in the entire TSS ± 1 kb region, whereas coverage was lowest at the TSS site (NFR; Fig. 4b).

Then we performed permutation tests to analyze whether these breast cancer–specific TSSs identified from the plasma cfDNA were expected based on expressed genes from the TCGA breast cancer data. We observed enrichment for lower coverage depth near TSSs in breast cancer patients compared to healthy donors for highly expressed genes in primary tumor tissue compared to the adjacent breast tissue (TCGA; *p* = 0.0030) but not for HTSSs of highly expressed genes (*p* = 0.0039; Fig. 4c). However, this effect was not significant when we compared coverage depth for lowly expressed genes in primary tumor tissue (TCGA) near TSSs in breast cancer patients versus healthy donors. This may be because of the difference between cfDNA from healthy individuals and from tumor-adjacent breast tissue.

### Different TSSs in pretreatment cfDNA between patients with breast cancer and healthy individuals

**Fig. 5:**
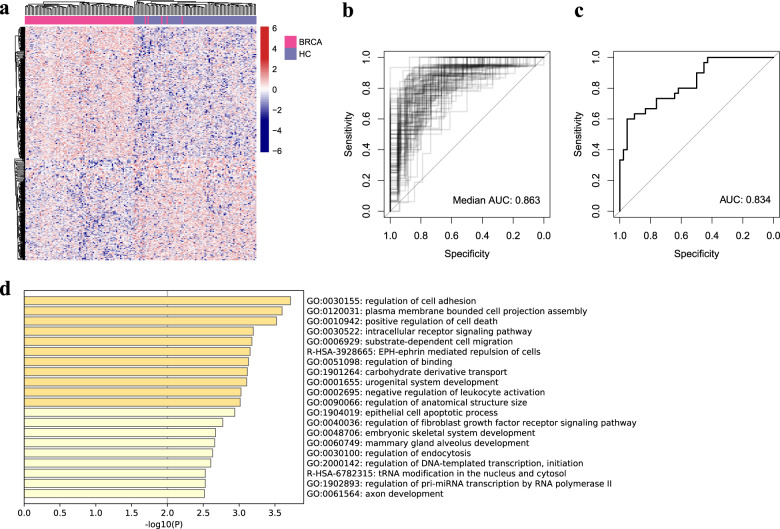
Prediction of breast cancer patients. **a** Heatmap of different TSS region coverage between patients with breast cancer and healthy donors. Receiver operating characteristic (ROC) curves for the classifier in training cohort (**b**) and in validation cohort (**c**). **d** The top 20 significantly different pathways between breast cancer and healthy donors.

We compared sequence coverage depth around cfDNA TSSs between patients with breast cancer and healthy donors. Technical reproducibility was evaluated using six samples, and the distance of each three technical replicates of the same sample was closer than those from different samples based on PCA analysis. (Supplementary Fig. 2). Among a total of 32444 tested TSSs, 414 TSSs were significantly different (*p* < 0.01, |log[fold change]| ≥ log1.2 and FDR < 0.1): 244 TSSs with relatively high coverage and 170 TSSs with relatively low coverage in patients with breast cancer (*n* = 85) compared with healthy donors (*n* = 85) (Supplementary Table S1a). Hierarchical clustering analyses showed an obvious separation of patients with breast cancer from healthy donors (Fig. 5a).

To assess the ability of coverage at TSS regions to classify individuals into cancer and healthy, we constructed LASSO classifier and repeated fivefold cross validation for 100 times to prevent biases. And we recollected 60 patients’ and 30 healthy donors’ plasma in an independent validation test. High values of the area under curve (AUC, median: 0.863 in training cohort; and 0.834 in validation cohort) were observed using receiver operating characteristic (ROC, Fig. 5b, c). The significantly different genes were related mainly to regulation of cell adhesion, positive regulation of cell death, etc (Fig. 5d), and the related genes were listed in Supplementary Table S1b.

Simultaneously, we compared TSS profiles between different tumor stage, ER status and molecular subtypes. And the results showed that the early stage (T1 and T2, Supplementary Fig. 3a; I and II, Supplementary Fig. 3b) and late stage (T3 and T4, Supplementary Fig. 3a; III and IV, Supplementary Fig. S3b) groups, ER positive and negative groups (Supplementary Fig. 4a), and different molecular subtypes (Supplementary Fig. 4b, c) could also be clustered into different groups, in particular luminal A vs triple negative subtype (Supplementary Fig. 4d). We also found some different related genes, such as cell adhesion related genes (*ITGBL1, RAPGEF4, ADGRL3, CDH18, DCAF6, CUTA*) in late-stage cancer group (Supplementary Table S2a, b, c), *BCAR1* in ER positive group (Supplementary Table S3a, b) and some genes (*ADCY2*, *CALM2*, *HSPA2*, *HSP90AA1*, *PIK3CA*, *AKT3* and *SHC4*) related to estrogen signaling pathway in luminal A group (Supplementary Tables S4a, b and S5a, b).

### Different TSSs in pretreatment cfDNA between responders and nonresponders

**Fig. 6:**
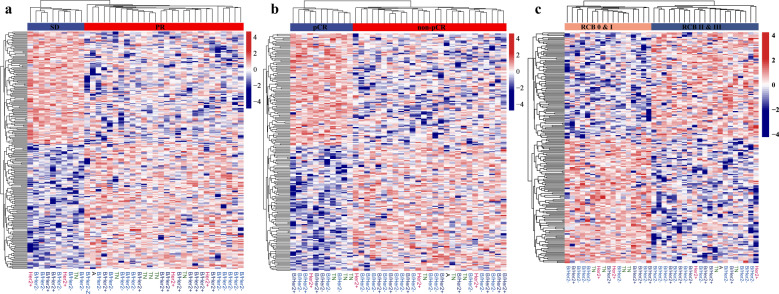
Different TSSs in pretreatment samples. Different TSSs between responders (PR) and nonresponders (SD) (**a**), between patients with pCR and npCR (**b**) and between patients with low RCB (RCB 0 & I) and high RCB (RCB II & III) by hierarchical clustering analyses. Red and blue represent relatively high and low coverage, respectively.

**Fig. 7:**
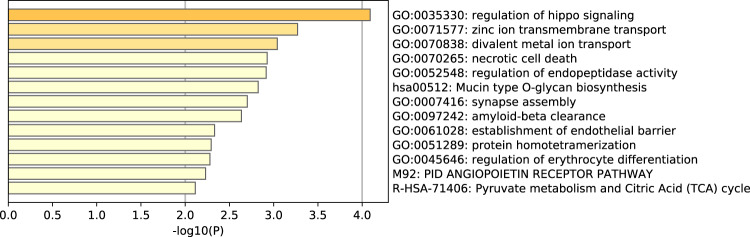
Top 13 significantly different pathways between responders and nonresponders in pretreatment samples.

We compared sequence coverage depth around TSSs of cfDNA between responders and nonresponders at pretreatment. A total of 232 TSS regions (*p* < 0.01 and |log[fold change]| ≥ log1.5) differed significantly in the 28 responders compared to the 10 nonresponders: 100 TSSs had high coverage and 132 TSSs had relatively low coverage in responders (Supplementary Table S6a). Hierarchical clustering analyses showed an obvious separation of responders from nonresponders (Fig. 6a). Gene functional annotation analyses revealed the top 13 pathways (Fig. 7, Supplementary Table S6b). Significantly differentially expressed genes included those involved in the regulation of hippo signaling, a pathway that inhibits cell growth (GO:0035330: NIMA related kinase 8 [*NEK8*], WT1 interacting protein [*WTIP*], WW and C2 domain containing 2 [*WWC2*], large tumor suppressor kinase 1 [*LATS1*]); necrotic cell death (GO:0070265: olfactomedin 4 [*OLFM4*], ring finger and FYVE-like domain containing E3 ubiquitin protein ligase [*RFFL*], spermatogenesis-associated 2 [*SPATA2*], forkhead box O3 [*FOXO3*], TNF receptor superfamily member 10c [*TNFRSF10C*], Rho/Rac guanine nucleotide exchange factor 2 [*ARHGEF2*], BCL2-like 2 [*BCL2L2*], CD70 molecule [*CD70*], tumor necrosis factor [*TNF*]); the intrinsic apoptotic signaling pathway in response to DNA damage (GO:0008630: *BCL2L2*, clusterin [*CLU*], *TNF*, apoptosis enhancing nuclease [*AEN*]); positive regulation of reactive oxygen species biosynthesis (GO:1903428: *CLU*, *FOXO3*, *TNF*); and the PID angiopoietin receptor pathway (M92: angiopoietin 2 [*ANGPT2*], CUGBP Elav-like family member 1 [*ELF1*], *TNF*). It is interesting that these genes had high TSS coverage in responders. However, several pathways were found in nonresponders, including pathways involved in pyruvate metabolism and the citric acid (TCA) cycle (R-HSA-71406) and protein O-linked glycosylation (GO:0006493). Significantly different pathways are listed in (Fig. 7, Supplementary Table S6b). Common pathways for each of the two groups are listed in Supplementary Table 6c, d.

We further compared patients with pCR and npCR, and with low RCB (residual cancer burden, RCB; RCB 0 and I) and high RCB (RCB II and III) at pretreatment, and hierarchical clustering analyses also showed an obvious separation between them (Fig. 6b, c). There were a total of 200 TSS regions differed significantly in the 11 patients with pCR compared to 27 patients with npCR: 95 TSSs with relatively high coverage in pCR patients, and 105 TSSs with relatively low coverage (Supplementary Table 7a), and 194 TSS regions differed significantly in the 17 patients with low RCB compared to 21 patients with high RCB: 102 TSSs with relatively high coverage in patients with low RCB, and 92 TSSs with relatively low coverage in patients with high RCB (Supplementary Table S7b) (*p* < 0.01 and |log[fold change]| ≥ log1.5). Gene functional annotation analyses showed that these genes was related to reveal the top 15 pathways, including regulation of PTEN gene transcription (R-HSA-8943724), PID INTEGRIN A481 PATHWAY (M277), positive regulation of cell cycle (GO:0045787) (Supplementary Fig. S5).

### Differently altered TSSs after neoadjuvant chemotherapy between responders and nonresponders

**Fig. 8:**
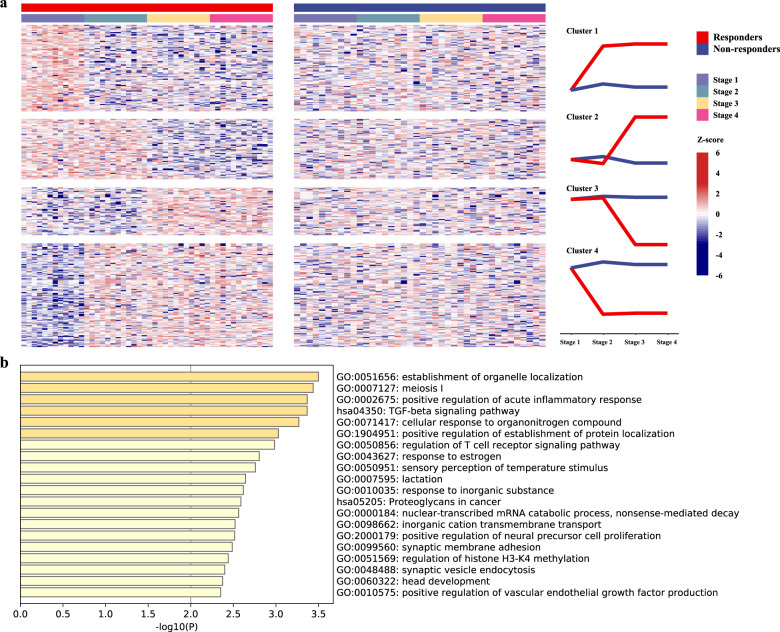
Differentially altered TSSs between responders and nonresponders after neoadjuvant chemotherapy. **a** Heatmaps summarizing changes in TSS region coverage over time in two major molecular functional groups in responders (PR) and nonresponders (SD). Samples are ordered from left to right by patient identification for each stage. Red and blue represent relatively high and low coverage, respectively. Stage 1 represents pretreatment, stage 2 represents post-1 cycle, stage 3 represents post-3/4 cycles, and stage 4 represents post-8 cycles. **b** The top 20 significantly altered pathways after neoadjuvant chemotherapy in responders. Red and blue represent relatively high and low coverage, respectively.

We analyzed paired plasma specimens before (pretreatment), during (post-1 cycle, post-3/4 cycles), and after (post-8 cycles) neoadjuvant chemotherapy to compare sequence coverage depth changes around TSSs in 12 responders and 10 nonresponders. The TSS regions of 321 genes (*p* < 0.01 and |log[fold change]| ≥ log1.5) were significantly differentially covered in responders: 93 of these genes’ TSS regions were downregulated and 112 were upregulated during early treatment (post-1 cycle), and 66 of these genes’ TSS regions were downregulated and 50 were upregulated during mid-treatment (post-3/4 cycles), with stable coverage after treatment (post-8 cycles). Conversely, these genes were not altered throughout the treatment period in nonresponders. Functional enrichment analyses revealed the top 20 pathways (Fig. 8a). Note that these significantly different genes were related mainly to positive regulation of the acute inflammatory response (GO:0002675), the TGF-beta signaling pathway (hsa04350), regulation of the T cell receptor signaling pathway (GO:0050856), the nuclear-transcribed mRNA catabolic process, nonsense-mediated decay (GO:0000184), and positive regulation of vascular endothelial growth factor production (GO:0010575) and proteoglycans in cancer (Fig. 8b). *BRCA1* (breast cancer 1 early onset), *HIC1* (HIC ZBTB transcriptional repressor 1), and *HMGB1* (high mobility group box 1) were involved in several of these pathways. Three pathways were related to cellular response to organonitrogen localization, response to estrogen, and lactation. Individual genes with significantly different coverage from these pathways are listed in Supplementary Table S8a. It is interesting that the 205 genes that were significantly altered from pretreatment to early treatment (post-1 cycle) in responders mainly comprised breast cancer 1 early onset [*BRCA1*], erb-b2 receptor tyrosine kinase 4 [*ERBB4*], GRB2-associated binding protein 1 (endothelial cell chemotaxis to vascular endothelial growth factor [*GAB1*]), mediator complex subunit 1 (epithelial cell proliferation involved in mammary gland duct elongation [*MED1*]), pro-apoptotic WT1 regulator (positive regulation of hydrogen peroxide-mediated programmed cell death [*PAWR*]), phosphatidylinositol-4,5-bisphosphate 3-kinase catalytic subunit gamma [*PIK3CG*], protein kinase D2 [*PRKD2*], and transforming growth factor beta 3 [*TGFB3*]. These genes were involved in positive regulation of the acute inflammatory response, positive regulation of vascular endothelial growth factor production, response to estrogen cellular response to growth factor stimulus, regulation of the T cell receptor signaling pathway, and positive regulation of response to DNA damage (Supplementary Table S8b). However, 116 genes that were significantly altered from pretreatment to mid-treatment (post-3/4 cycles) in the responders were involved mainly in the PID TGFBR pathway, nonsense-mediated decay, and the establishment of mitotic spindle orientation (Supplementary Table S8c).

## Discussion

Previous studies have focused mainly on the relationship between ctDNA and cancer occurrence and development, relapse, metastasis, and drug resistance. ctDNA may be used as a biomarker for cancer screening, early diagnosis, individualized treatment, and prognostic evaluation based on the detection of CNVs^18^, mutations^4^, or methylation patterns^19^. However, the clinical utility of the cfDNA nucleosome footprint has not yet been fully confirmed. We provide new insight into the nucleosome footprint of plasma circulating cfDNA. Our work directly maps the nucleosome footprint of cell-free DNA.

To confirm the relationship between the cfDNA nucleosome footprint and gene expression, we performed correlation analyses among the nucleosome footprint of DNA in the cell supernatant, intracellular nucleosome positioning, and gene expression. The results showed that the length of cell supernatant DNA was similar to the length of DNA bound to the mononucleosome. Correlation analyses also confirmed the relationships among TSS coverage in cell supernatant DNA, intracellular nucleosome positioning, and gene expression (Fig. 3f, h). As expected, the TSS region coverage of cell supernatant DNA was positively correlated with intracellular nucleosome positioning and negatively correlated with gene expression. Nucleosome positioning relative to transcription start sites is directly correlated with RNA Pol II binding^10^, and transcriptionally active gene promoters are characterized by the presence of a NFR or NDR in their core region^9^. Therefore, we may infer that nucleosome footprint changes in vivo lead to gene expression or silencing.

DNA protected by nucleosomes is released into the bloodstream as cfDNA, which can be sequenced directly. It is interesting that cfDNA from patients is more closely related to gene expression in breast cancer cell lines than cell supernatant DNA, possibly because of the high similarity between bovine serum DNA in culture medium and human serum DNA or because of extracellular release without complete digestion, which affects analyses of cell supernatant DNA. A correlation between cfDNA and gene expression has been reported in previous studies^14^, but the gene expression data sets used were from public databases. In the current study, the correlation between cfDNA and nucleosome positioning and the correlation between cfDNA and gene expression were demonstrated more clearly as a result of the use of cell lines.

We also confirmed the association between the cfDNA nucleosome positioning in breast cancer patients and the expressed breast cancer-specific genes using TCGA breast cancer data (Fig. 4c). Parallel analysis with RNAseq and MNase-seq of the matched primary tumor and blood samples may facilitate the discovery of correlations between cfDNA nucleosome positioning and relevant gene expression with the nucleosome occupancy of the genes. cfDNA contains DNA from both normal and tumor tissues in patients with breast cancer, and studies have found cfDNA derived from tissue-specific and tumor-specific open chromatin regions (NFR or NDR)^20,21^. Because the fractions of tumor- and non-tumor cfDNA vary among different patients^4^, a limitation of our study is that we failed to consider the two fractions of normal and tumor DNA. Normalization of the tumor fractions may increase the cancer prediction accuracy. Another limitation of our study is that we failed to profile the nucleosome positioning of immune cells, which are the major components of non-tumor-derived normal DNA in patients with cancer^22,23^. MNase-seq of different immune cell types, as well as single-cell RNA sequencing of peripheral blood mononuclear and tumor cells, will help to further elucidate the contributions of tissues and the origins of cfDNA to better understand the complexity and heterogeneous nature of cfDNAs in patients with breast cancer.

For breast cancer, neoadjuvant chemotherapy is equivalent to postoperative treatment for breast cancer and is used to reduce tumor size, decrease tumor stage, prolong patients’ DFS and OS, and conserve breast tissue^24^. The decision to perform neoadjuvant chemotherapy for breast cancer is based on molecular typing; some patients do not benefit from this type of therapy. The response to neoadjuvant chemotherapy is usually assessed only after several treatment cycles, which leads to wasted resources and overtreatment of patients. Thus, another aim of the present study was to explore whether a characteristic plasma cfDNA profile can be used to predict the efficacy of neoadjuvant chemotherapy. We first analyzed pretreatment specimens and identified a number of biologic pathways related to treatment response, including regulation of hippo signaling, necrotic cell death, intrinsic apoptotic signaling pathway in response to DNA damage, and positive regulation of reactive oxygen species biosynthesis. Using Affymetrix GeneChip detection technology to analyze biopsy tissue, Larissa et al.^24^ identified biologic pathways related to docetaxel and capecitabine treatment, including spindle regulation and microtubule depolymerization, DNA repair, and cellular proliferation. Using PCR, Gianni and colleagues^25^ identified 86 genes that correlate with responsiveness to neoadjuvant doxorubicin and paclitaxel; these genes were from functional categories that influence sensitivity or resistance to chemotherapy (i.e., apoptosis, invasion, metastasis, drug resistance/metabolism, proliferation, ER). Ayers et al.^26^ built a 74-gene model classifier to predict pathologic response to neoadjuvant T/FAC therapy, achieving high positive predictive value and specificity.

Although the genes identified in the present study are different from those in previous reports, they belong to several of the same pathways (e.g., necrotic cell death and intrinsic apoptotic signaling in response to DNA damage). This may be explained by the fact that different genes play a role in different body parts. We also identified a pathway related to the regulation of erythrocyte differentiation, which may have been due to the presence of peripheral blood cell DNA; its relationship with tumorigenesis is unknown. These results support the feasibility of predicting the efficacy of neoadjuvant chemotherapy prior to treating breast cancer.

We examined changes in TSS region coverage in plasma collected from breast tumors at different time points during neoadjuvant chemotherapy as well as their association with response to treatment. Examining changes throughout treatment may provide more information regarding patient responsiveness than analyzing static time points. Analyzing changes throughout treatment may facilitate the development of improved predictors of response and drug resistance.

These altered genes were associated mainly with positive regulation of the acute inflammatory response, the TGF-beta signaling pathway, regulation of the T cell receptor signaling pathway, and positive regulation of vascular endothelial growth factor production (Fig. 6b). *BRCA1*, *HIC1*, and *HMGB1* were involved in several of these pathways. *BRCA1* is a tumor suppressor gene whose structural and functional abnormalities are closely related to the incidence of breast cancer. It plays an important role in the regulation of cell cycle progression, DNA damage, the repair of cell growth and apoptosis, transcriptional activation and inhibition, and other biological pathways^27^. *HIC1*, a tumor suppressor gene, is epigenetically silenced in a variety of tumors, and deleting *HIC1* might contribute to premalignant transformation in the early stages of tumor formation^28^. The *HMGB* family is a group of chromosomal proteins involved in DNA replication, recombination, transcription, and repair that is related to the progression of a variety of cancers, including colorectal cancer^29^, hepatocellular carcinoma^30^, and gastric cancer^31,32^. In recent years, an increasing number of studies have focused on changes in gene expression in serial biopsy tissue specimens. Genetic changes associated with prediction and prognosis involve the immune response^33–35^, cell proliferation^24,33–36^, apoptosis^34,35^, DNA repair^24^, and the antiinflammatory response^26^. These findings are consistent with those of the current study.

We also noted significantly altered genes in early treatment (post-1 cycle) in responders. These genes were involved in positive regulation of the acute inflammatory response, positive regulation of vascular endothelial growth factor production, response to estrogen, cellular response to growth factor stimulus, regulation of the T cell receptor signaling pathway, and positive regulation of response to DNA damage. Genes that were significantly altered mid-treatment (post-3/4 cycles) were involved in the PID TGFBR pathway, nonsense-mediated decay, and the establishment of mitotic spindle orientation. These results indicate that different gene changes occurred during chemotherapy treatment. It is also possible that gene expression was delayed for a period after nucleosomes were depleted at the transcription initiation sites^37^.

cfDNA in plasma comes from apoptotic cells and includes ctDNA released by tumor cells as well as DNA from peripheral blood cells and other tissue. Therefore, gene changes based on cfDNA analyses reflect not only tumor tissue but also the reactions of the blood system and other tissue in the body, such as immune cells. Another limitation of this study is its relatively small sample size. Therefore, we consider our analyses to be exploratory: Larger studies are required to validate our findings and confirm specific associations between molecular data and clinical outcomes.

In summary, we confirmed a correlation between the cfDNA nucleosome footprint profile in the region around TSSs and gene expression. We also found significantly different nucleosome footprint profiles in the region near TSSs in plasma cfDNA from healthy individuals versus patients with breast cancer and in plasma cfDNA from responders versus nonresponders before, during, and after a series of neoadjuvant chemotherapy treatment cycles. These genes were related to pathways involved in the inhibition of cell proliferation, response to DNA damage, and immune response. These findings are expected to increase the feasibility of plasma cfDNA nucleosome profiling as a new biomarker for predicting the efficacy of neoadjuvant chemotherapy for breast cancer.

## Methods

### Cell culture

The human breast cancer cell lines T-47D and MDA-MB-231 were obtained from ATCC. T-47D cells were cultured in RPMI-1640 medium (Gibco, Carlsbad, CA, USA) supplemented with 20 mM HEPES and 10% fetal bovine serum (FBS) at 37 °C in a humidified 5% CO_2_ atmosphere. MDA-MB-231 cells were cultured in DMEM medium (Gibco) supplemented with 20 mM HEPES and 10% FBS at 37 °C in a humidified 5% CO_2_ atmosphere. Cell supernatant and cell particles were collected over a period of 48 h and subsequently used for high-throughput sequencing.

### Patients and samples

The study included 85 healthy individuals from Guangzhou Darui Biotechnology company and 30 from the Third Affiliated Hospital of Southern Medical University, and 145 breast cancer patients from the First People’s Hospital of Foshan in Guangdong, China. Ethical approval for the study was received from the Ethics Committee of the Affiliated Foshan Hospital of Sun Yat-Sen University. All participants provided written informed consent. A total of 115 healthy individuals and 145 breast cancer patients were sampled before any treatment. 38 of 145 breast cancer patients received 24 weeks of sequential epirubicin-cyclophosphamide-docetaxel preoperative chemotherapy followed by resection. Of these 38 patients with neoadjuvant chemotherapy, 22 patients were also sampled at 3 time points during treatment: after the first cycle (post-1 cycle), after the third or fourth cycle (post-3/4 cycles; 10 patients post-3 cycles and 12 patients post-4 cycles), and after the eighth cycle (post-8 cycles). Postsurgical assessment was performed according to the evaluation criteria of RECIST (Response Evaluation Criteria In Solid Tumor) version 1.0^38^. Clinical characteristics of the patients are presented in Tables 1 and 2.

**Table 1:** Clinical characteristics of the 115 healthy individuals and 145 breast cancer patients in the study.

	Number of patients in training group	Number of patients in validation group
Healthy individuals	85	30
Age, mean (range), years	27.8 (19–45)	25.7 (27–48)
Sex, female	–	–
Breast cancer patients	85	60
Age, mean (range), years	50.6 (32–88)	49.6 (26–83)
Sex, female	85	–
Tumor staging		
Tis/T1/T2//T3/T4	0/19/48/5/13	1/22/36/0/1
Node staging		
N0/N1/N2/N3	31/41/9/4	37/20/2/1
Metastasis staging		
M0/M1	85/0	59/1
Combined staging		
0/IA/IIA/IIB	0/13/22/25	1/16/25/13
IIIA/IIIB/IIIC/IV	10/10/4/1	2/1/1/1
Subtype		
HR+Her2-	47	47
HR+Her2+	13	12
HR-Her2+	6	1
HR-Her2-	19	0
Luminal A	26	12
Luminal B	34	47
Her2	6	1
Triple negative	19	0

*Subtype* immunohistochemically categorized subtype.

**Table 2:** Clinical characteristics of the 38 breast cancer patients undergoing neoadjuvant chemotherapy in the study.

	Number of patients
Age, mean (range), years	48.0 (34–65)
Sex, female	38
Tumor staging	
T1/T2//T3/T4	4/28/1/5/
Node staging	
N0/N1/N2/N3	11/23/2/2
Metastasis staging	
M0/M1	38/0
EC-T	34
T-EC	4
Tumor response	
PR	28
SD	10
pCR (RCB 0)	11
npCR	27
RCB-I	6
RCB-II	15
RCB-III	6
Subtype	
Luminal A	1
Luminal B	28
Her2	6
Triple negative	3

*Subtype* immunohistochemically categorized subtype, *E* epirubicin, *C* cyclophosphamide, *T* docetaxel, *EC-T* (epirubicin-cyclophosphamide) × 4 cycles + docetaxel × 4 (3 weeks per cycle), *T-EC* docetaxel × 4 + (epirubicin-cyclophosphamide) × 4 (3 weeks per cycle), *PR* partial response, *SD* stable disease, *pCR* pathologic complete response, *npCR* none pathologic complete response, *RCB* residual cancer burden.

### cfDNA sequencing

A total of 1 mL peripheral blood was collected in EDTA tubes from each patient and immediately centrifuged for 10 min at 16,000 rpm, 4 °C, and ~500 µL plasma and cell supernatant was stored at −80°C before use, which yielded at least 1 ng total cfDNA for sequencing. cfDNA extraction from plasma and cell supernatant was performed with the QIAamp DNA Blood Mini Kit (Qiagen). We prepared a starting amount of approximately 1–5 ng DNA (three biological replicates per input for six samples) for library construction using the Life Sciences Ion Xpress™ Plus Fragment Library Kit, and we omitted the fragmentation step because of the degradation of plasma DNA. The number of PCR cycles was set to 12. Libraries were analyzed on a Bioanalyzer instrument (Agilent Technologies, Singapore) to observe the DNA size distribution. Sequencing was performed with the Ion PI™ Hi-Q™ OT2 200 Kit and the Ion PI™ Hi-Q™ Sequencing 200 Kit. Ten libraries were pooled together and subjected to 520 flow on the Ion Proton platform (ThermoFisher Scientific, USA), and 6–10 million reads were generated for each cfDNA sample.

### MNase sequencing

Approximately 10^7^ cells were prepared for nucleosome digestion and DNA extraction with the Active Motif Inc Nucleosome Preparation Kit. A total of 50 µL obtained chromatin and 2.5 µL working stock enzyme was incubated for 15 min at 37 °C. The digested nucleosome samples were immediately used for the next step of DNA extraction. Approximately 100 ng DNA was used for library construction and sequencing. Libraries were analyzed on a Bioanalyzer instrument (Agilent Technologies) to observe the DNA size distribution. The kits and parameters used were the same as for cfDNA sequencing. Approximately 100 million reads were generated per sample by MNase-seq.

### Gene expression sequencing (mRNA sequencing)

We extracted RNA from approximately 10^7^ cell particles using TRIzol Reagent (Invitrogen, USA). The amount and quality of the RNA were assessed with a NanoDrop™ 8000 UV Spectrophotometer (Thermo Scientific, USA). We used 1 µg total RNA for mRNA purification using a Dynabeads™ mRNA DIRECT™ Purification Kit (Invitrogen). We prepared the purified product for library construction using the Ion Total RNA-Seq Kit v2. We quantified the concentration of mRNA using the Qubit™ 3.0 Fluorometer (Invitrogen). Experimental operations followed the RNA enrichment and library generation protocols provided in the manual. Two libraries were pooled together and subjected to 520 flow on the Ion Proton platform (ThermoFisher Scientific), and 30–40 million reads per sample were generated.

### Sequencing read alignment and processing

For cfDNA sequencing and MNase sequencing, we aligned sequencing reads with the human reference genome (hg19) using TMAP and removed PCR duplicates using the SAMtools (version 1.9) rmdup function^39^. For mRNA sequencing, we aligned sequencing reads with the GENCODE human transcriptome (Release 30) using Salmon (version 0.13.1)^40^ and used transcripts per million (TPM) to quantify the expression of each gene.

### TSS profiles of cDNA and MNase sequencing

Gene information was obtained from RefSeq. For cfDNA sequencing data, we calculated read counts of regions ranging from –1k bp to +1k bp around TSSs using bedtools (version 2.17.0), then normalized them using the reads per kilobase per million mapped reads (RPKM) method to present cfDNA-based nucleosome occupancy. In the MNase sequencing data, only the nucleosome-depleted region (NDR; from –150 bp to +50 bp of the TSS) showed depleted coverage, so we used coverage depth of the NDR to quantify the nucleosome occupancy of each TSS. The total depth of each NDR was calculated with SAMtools (version 1.9).

Based on the cfDNA RPKM value of each TSS, we performed Wilcoxon rank sum tests (two-sided) to identify TSSs with altered cfDNA coverage between groups. TSSs with *p* < 0.01 and |log(fold change)| ≥ log1.5 were considered significantly changed. Hierarchical clustering was performed with the average linkage clustering algorithm, and heatmaps were plotted with the pheatmap package (version 1.0.12).

### Correlation analyses of cfDNA-seq, MNase-seq, and RNA-seq data

To evaluate concordance across the three platforms, we performed Spearman’s rank correlation analyses for gene expression, MNase-based TSS nucleosome occupancy, and cfDNA-based TSS nucleosome occupancy profiles. Moreover, for MNase-seq and RNA-seq data, sequence coverage depth around TSSs was compared between highly expressed genes (TPM > 10) and unexpressed genes (TPM = 0), and the depth of each genomic site was calculated with SAMtools (version 1.9).

Furthermore, we performed permutation tests to estimate the significance of overlaps between gene expression profiles and TSS profiles (cfDNA-seq and MNase-seq, respectively). We computed the frequency of highly expressed genes (TPM > 10) with high nucleosome occupancy (genes in the upper quartile), unexpressed genes (TPM = 0) with high nucleosome occupancy, highly expressed genes with low nucleosome occupancy (genes in the last quartile), and unexpressed genes with low nucleosome occupancy. A null distribution was generated from 1,000 permutations. The distributions were then standardized based on *z* scores and used to compute two-sided *p* values to determine the significance of overlaps.

Similarly, we estimated the concordance between differentially expressed genes and genes with altered cfDNA TSS coverage in breast cancer patients. We filtered the list of differentially expressed genes using GEPIA (http://gepia.cancer-pku.cn)^41^, and genes with different cfDNA TSS coverage (*p* < 0.01 and |log[fold change]| ≥ log1.5) between breast cancer patients and healthy donors were selected. Permutation tests were performed to determine the significance of overlaps.

### Technical reproducibility assessment

Technical reproducibility of TSS coverage between replicates using Principal Component Analysis (PCA).

### Procedure of classifiers construction

Genes with significant differential TSS coverages were used to develop promoter profiling-based classifiers, and fivefold cross validation was used to randomly divide samples into training and validation sets and evaluate the performance. In the training set, the normalized read count of each TSS was discretized according to the optimal cut-off point before classifier construction. The optimal cut-off point of each promoter was defined as the maximum value of (sensitivity + specificity)/2 in the training sets. R package glmnet (version 2.0–16) was used to perform the least absolute shrinkage and selection operator (LASSO). Receiver operating characteristic (ROC) analysis was used to calculate area under curve (AUC) of the validation set using pROC (version 1.16.2) R package (version 3.5.1). The whole process was repeated 100 times.

### Functional annotation and enrichment

We performed functional annotation and enrichment analyses using metascape (http://metascape.org)^42^.

### Reporting summary

Further information on research design is available in the Nature Research Reporting Summary linked to this article.

## Supplementary information

Supplementary information

Supplementary Data files

Reporting Summary Checklist

## Data Availability

All novel sequencing datasets (cfDNA whole-genome sequencing, MNase sequencing and mRNA sequencing) generated during this study, are publicly available in Sequence Read Archive: https://identifiers.org/ncbi/insdc.sra:SRP302308^43^. All other datasets generated and analyzed during the study, are available in the figshare repository: 10.6084/m9.figshare.13709953^44^. Data supporting Fig. 5a, d and Figs. 6–8, are included in the supplementary tables that accompany the article. The data generated and analyzed during this study are described in the following metadata record: 10.6084/m9.figshare.13738795^45^.

## Code availability

Relevant custom code is available at https://github.com/hanbw0120/cfDNA_breast_cancer.
