# Supplementary material for: Association between the nucleosome footprint of plasma DNA and neoadjuvant chemotherapy response for breast cancer
Source: NPJ Breast Cancer. 2021 Mar 26;7:35. doi: 10.1038/s41523-021-00237-5 (PMC7997954; doi:10.1038/s41523-021-00237-5)
Supplement: Supplementary file 1 — Supplementary information [file 41523_2021_237_MOESM1_ESM.pdf]

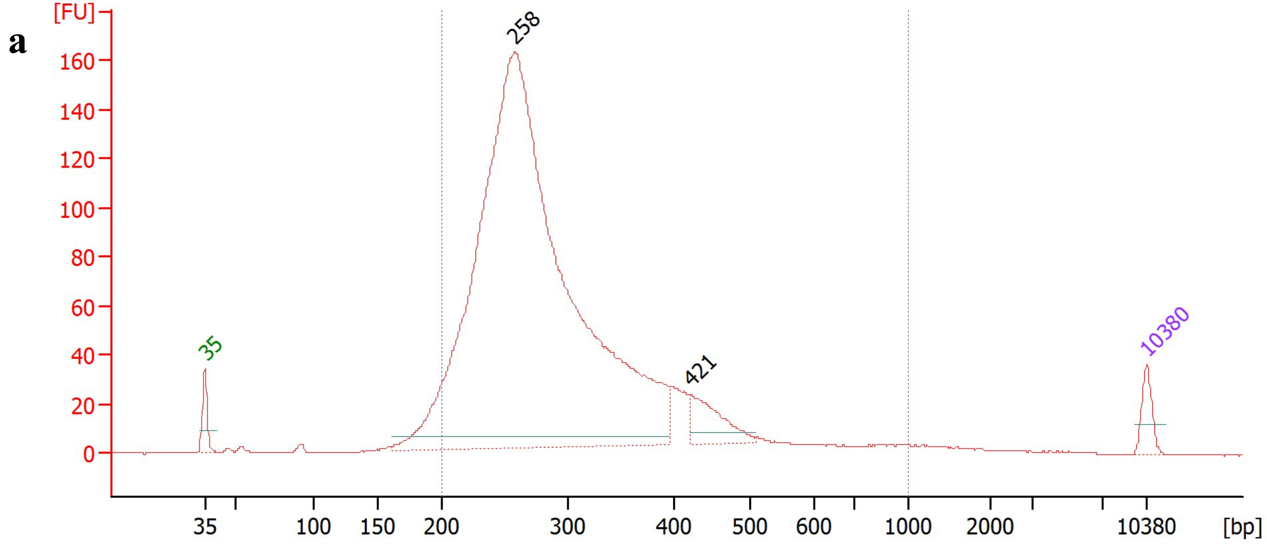

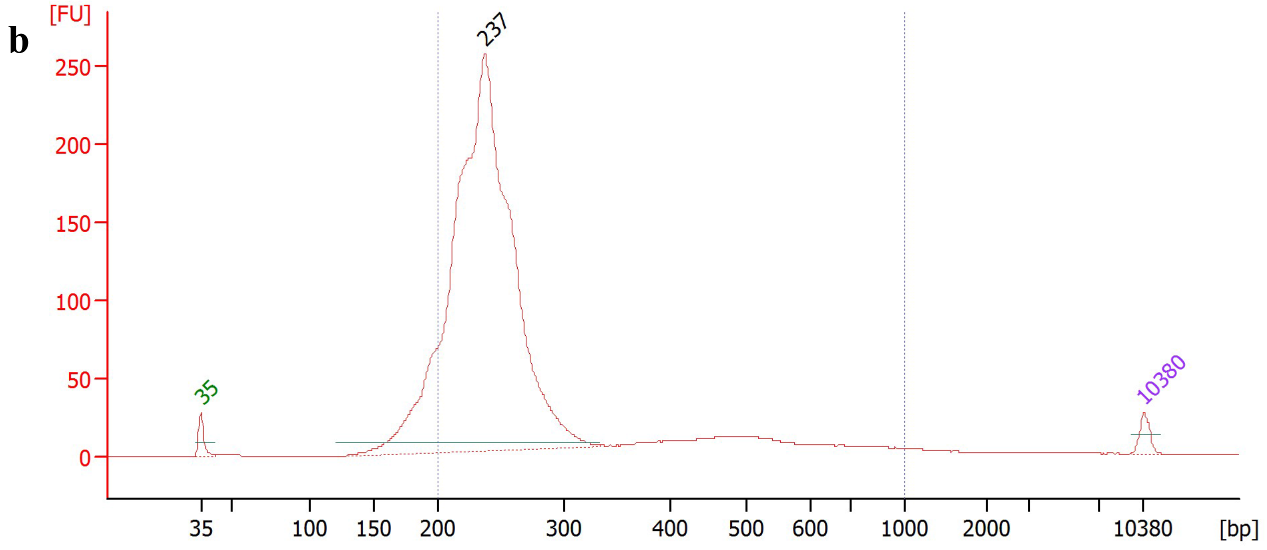

Supplementary Figure 1. Size distribution of cfDNA library of cell supernatant (a) and intracellular MNase-digestion genome DNA library (b).

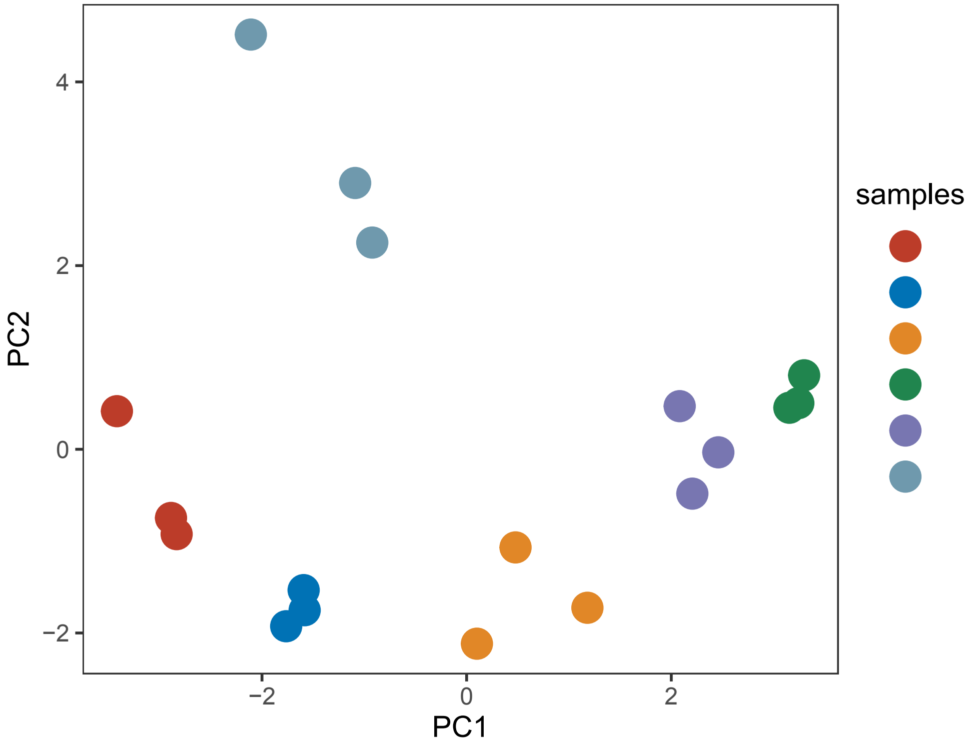

Supplementary Figure 2. PCA analysis of genome-wide cfDNA-seq signal from 6 breast cancer patients (3 biological replicates per sample).

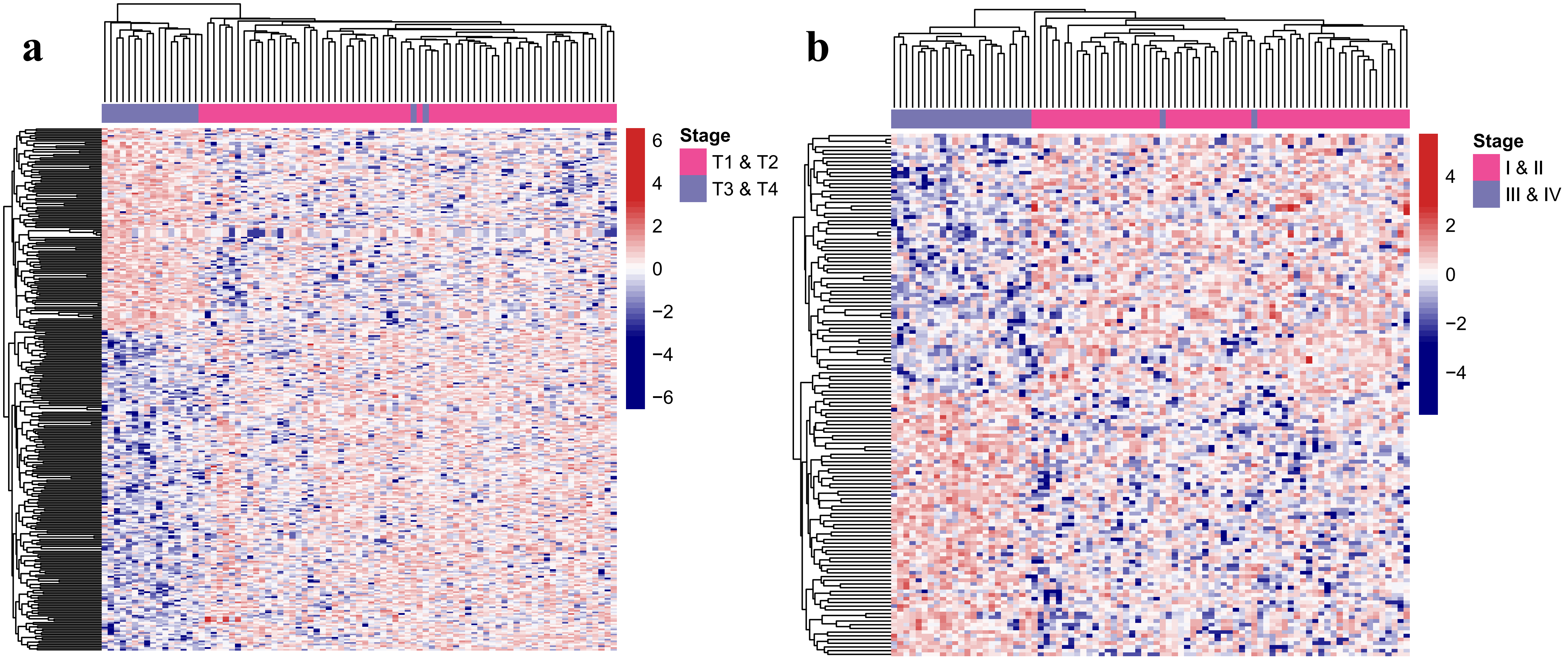

Supplementary Figure 3. Heatmap of different TSS region coverage between early stage (T1 and T2) patients and late stage (T3 and T4) patients (a), and between early stage (I and II) patients and late stage (III and IV) patients (b).

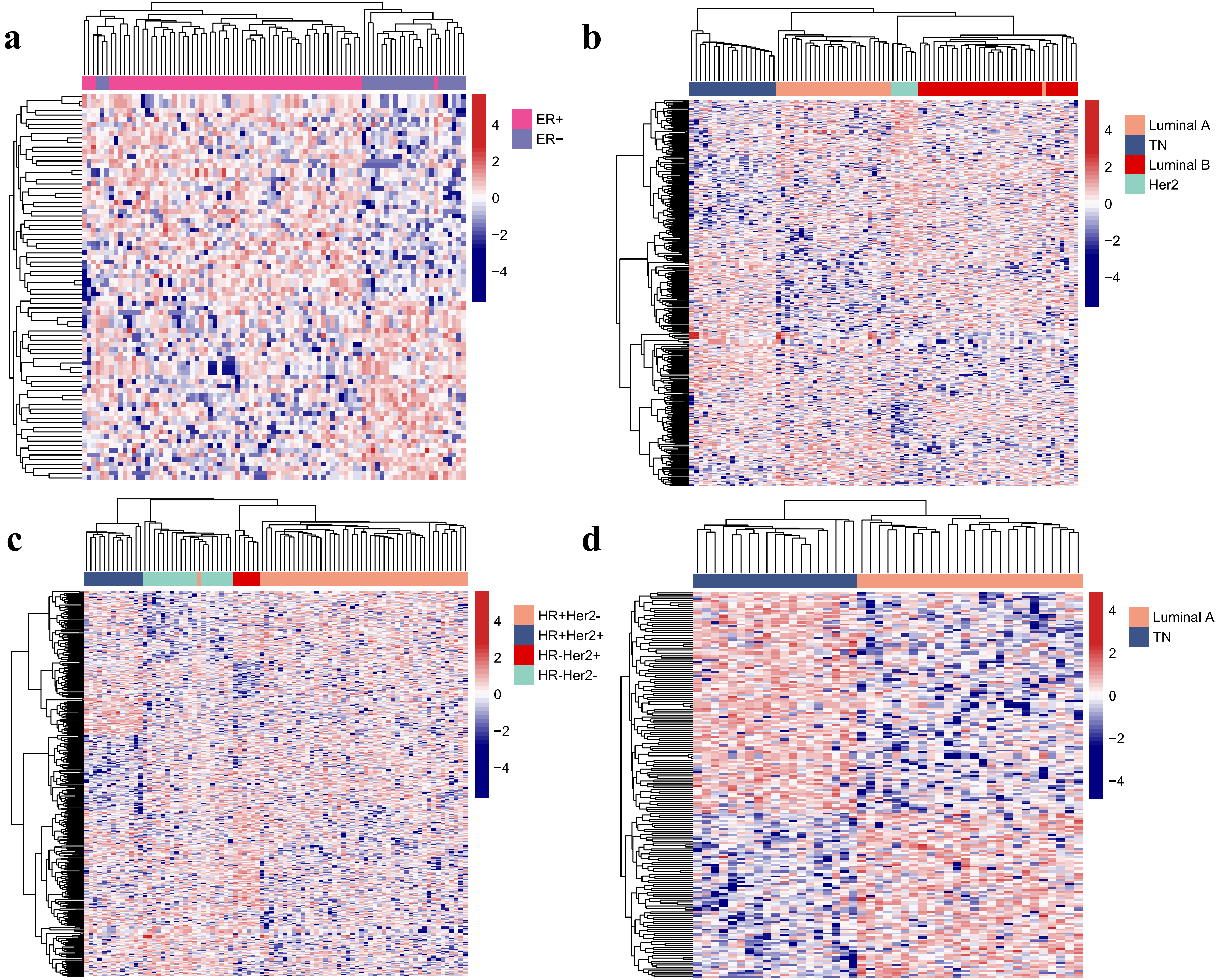

Supplementary Figure 4. Heatmap of different TSS region coverage between ER+ and ER- (a), different molecular subtypes (b, c), and luminal A and triple negative subtype (d).

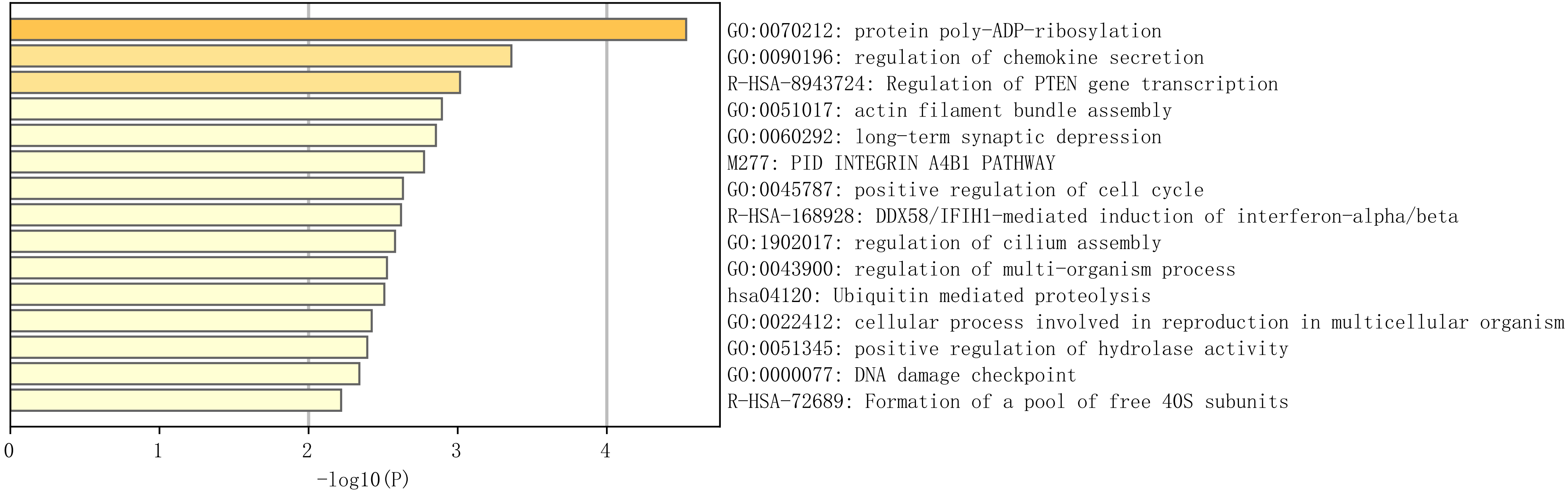

Supplementary Figure 5. The top 15 pathways between patients with pCR and npCR.

Supplementary Data Set

Supplementary Tables 1-8 could be found in Supplementary data.
