# Supplementary material for: Association between the nucleosome footprint of plasma DNA and neoadjuvant chemotherapy response for breast cancer
Source: NPJ Breast Cancer. 2021 Mar 26;7:35. doi: 10.1038/s41523-021-00237-5 (PMC7997954; doi:10.1038/s41523-021-00237-5)
Supplement: Supplementary file 3 — Reporting Summary Checklist [file 41523_2021_237_MOESM3_ESM.pdf]

nature research

Corresponding author(s): Xin Li, Guo-Lin Ye, Xue-Xi Yang

Last updated by author(s): Nov 3, 2020

# Reporting Summary

Nature Research wishes to improve the reproducibility of the work that we publish. This form provides structure for consistency and transparency in reporting. For further information on Nature Research policies, see our Editorial Policies and the Editorial Policy Checklist.

## Statistics

For all statistical analyses, confirm that the following items are present in the figure legend, table legend, main text, or Methods section.

| n/a | Confirmed | |
|---|---|---|
| ☐ | ☒ | The exact sample size (*n*) for each experimental group/condition, given as a discrete number and unit of measurement |
| ☐ | ☒ | A statement on whether measurements were taken from distinct samples or whether the same sample was measured repeatedly |
| ☐ | ☒ | The statistical test(s) used AND whether they are one- or two-sided<br>*Only common tests should be described solely by name; describe more complex techniques in the Methods section.* |
| ☒ | ☐ | A description of all covariates tested |
| ☐ | ☒ | A description of any assumptions or corrections, such as tests of normality and adjustment for multiple comparisons |
| ☐ | ☒ | A full description of the statistical parameters including central tendency (e.g. means) or other basic estimates (e.g. regression coefficient) AND variation (e.g. standard deviation) or associated estimates of uncertainty (e.g. confidence intervals) |
| ☐ | ☒ | For null hypothesis testing, the test statistic (e.g. *F*, *t*, *r*) with confidence intervals, effect sizes, degrees of freedom and *P* value noted<br>*Give P values as exact values whenever suitable.* |
| ☒ | ☐ | For Bayesian analysis, information on the choice of priors and Markov chain Monte Carlo settings |
| ☒ | ☐ | For hierarchical and complex designs, identification of the appropriate level for tests and full reporting of outcomes |
| ☐ | ☒ | Estimates of effect sizes (e.g. Cohen's *d*, Pearson's *r*), indicating how they were calculated |

*Our web collection on statistics for biologists contains articles on many of the points above.*

## Software and code

Policy information about availability of computer code

| | |
|---|---|
| Data collection | Sequencing data collection was carried out using Ion Proton platform (ThermoFisher Scientific, USA). |
| Data analysis | Open source software used for data analysis: R (version 3.5.2), Python (version 2.7), SAMtools (version 1.9), bedtools (version 2.17), and TMAP. Functional annotation and enrichment were performed using metascape. Prediction was implemented using R package glmnet (version 2.0) and pROC (version 1.15). Graphics were generated using R package ggplot2 (version 3.2) and ggsci (version 2.9). |

For manuscripts utilizing custom algorithms or software that are central to the research but not yet described in published literature, software must be made available to editors and reviewers. We strongly encourage code deposition in a community repository (e.g. GitHub). See the Nature Research guidelines for submitting code & software for further information.

## Data

Policy information about availability of data

All manuscripts must include a data availability statement. This statement should provide the following information, where applicable:

- Accession codes, unique identifiers, or web links for publicly available datasets
- A list of figures that have associated raw data
- A description of any restrictions on data availability

The aligned reads of the merged samples reported in this paper have been deposited at the database of NODE (https://www.biosino.org/node, project ID: OEP000713).

# Field-specific reporting

Please select the one below that is the best fit for your research. If you are not sure, read the appropriate sections before making your selection.

☒ Life sciences ☐ Behavioural & social sciences ☐ Ecological, evolutionary & environmental sciences

For a reference copy of the document with all sections, see nature.com/documents/nr-reporting-summary-flat.pdf

# Life sciences study design

All studies must disclose on these points even when the disclosure is negative.

| | |
|---|---|
| Sample size | 85 healthy donors vs. 85 breast cancer patients, 28 responders vs. 10 nonresponders to neoadjuvant chemotherapy |
| Data exclusions | No data were excluded from the analyses. |
| Replication | Plasma cfDNA from 6 patient samples was tested 3 times, and other assays were performed only once per sample. |
| Randomization | All human participants belonged to pre-defined groupings based on the cancer type. With regards to the cell lines' experiments, samples were not randomized, since there was no experimental groups. There was no control for other covariates since each cell line was compared against itself |
| Blinding | Plasma samples were blinded during the sample preparation and sequencing. Data analysis was performed unblinded on the discovery cohort and blinded on the validation cohort. |

# Reporting for specific materials, systems and methods

We require information from authors about some types of materials, experimental systems and methods used in many studies. Here, indicate whether each material, system or method listed is relevant to your study. If you are not sure if a list item applies to your research, read the appropriate section before selecting a response.

## Materials & experimental systems

| n/a | Involved in the study | |
|---|---|---|
| ☒ | ☐ | Antibodies |
| ☐ | ☒ | Eukaryotic cell lines |
| ☒ | ☐ | Palaeontology and archaeology |
| ☒ | ☐ | Animals and other organisms |
| ☐ | ☒ | Human research participants |
| ☒ | ☐ | Clinical data |
| ☒ | ☐ | Dual use research of concern |

## Methods

| n/a | Involved in the study | |
|---|---|---|
| ☒ | ☐ | ChIP-seq |
| ☒ | ☐ | Flow cytometry |
| ☒ | ☐ | MRI-based neuroimaging |

## Eukaryotic cell lines

Policy information about cell lines

| | |
|---|---|
| Cell line source(s) | T-47D and MDA-MB-231 cell lines obtained from ATCC |
| Authentication | ATCC standard protocols - Human STR Profiling Cell Authentication |
| Mycoplasma contamination | mycoplasma free |
| Commonly misidentified lines (See ICLAC register) | No commonly misidentified cell lines were used. |

## Human research participants

Policy information about studies involving human research participants

| | |
|---|---|
| Population characteristics | Population characteristics for study participants can be found in Tables 1. In brief, 85 healthy controls and 85 breast cancer patients were included, 28 responders and 10 nonresponders before, and 12 res. and 10 nonres. after neoadjuvant chemotherapy were tested and analyzed. All the participants were females. |
| Recruitment | Plasma samples from 85 healthy individuals were collected from Guangzhou Darui Biotechnology company, and 85 breast cancer patients from the First People's Hospital of Foshan in Guangdong, China. Potential self-selection bias or other biases |

| | |
|---|---|
| | were not identified. |
| Ethics oversight | All patients have provided written informed consents, and all samples have been obtained upon approval of the institutional ethics committees (the Ethics Committee of the Affiliated Foshan Hospital of Sun Yat-Sen University). |

Note that full information on the approval of the study protocol must also be provided in the manuscript.
